# Exploring Client-Centered Care Experiences in In-Patient Rehabilitation Settings

**DOI:** 10.1177/2333393615582036

**Published:** 2015-04-15

**Authors:** Elena L. Bamm, Peter Rosenbaum, Seanne Wilkins, Paul Stratford, Nadilein Mahlberg

**Affiliations:** 1 McMaster University, Hamilton, Ontario, Canada

**Keywords:** client-centered care, quality of care, relationships, patient-provider, stroke, partnerships

## Abstract

Patient or client-centered care has been widely accepted as an essential component of health care delivery in many countries. Few studies explore actual implementation of client-centered principles and clients’ and health care providers’ (HCPs) experiences with these approaches. Our objective was to explore current models of delivery of rehabilitation services from the perspectives of patients, families, and HCPs. We conducted semistructured interviews with patients, families, and HCPs of one of four rehabilitation facilities in South-Central Ontario, Canada. *Being on common grounds/Working toward client set goals* was the main category identified by both clients and HCPs. Although successful partnerships were created, the majority of clients assumed a passive position. Clients needed more information about the rehabilitation progression and alternative treatment options. The results of the study suggest that we need to encourage and educate clients to become motivated, well-informed, proactive participants in their care.

Patient or client-centered care (CCC) has been widely accepted as an essential component of health care delivery in many countries. Thousands of articles have been published on the subject in the last decade. Similar to any developing subject, there are many names (e.g., person-, patient-, client-, family-centered care) and even more new definitions and conceptualizations of this idea (Abdelhadi & Drach-Zahavy, 2012; Bamm & Rosenbaum, 2008; Bechtel & Ness, 2010; Bertakis & Azari, 2011). Although they might differ slightly in how the concepts are operationalized, essentially all describe care

. . . that establishes a partnership among practitioners, patients, and their families (when appropriate) to ensure that decisions respect patients’ wants, needs, and preferences and that patients have the education and support they need to make decisions and participate in their own care. (National Research Council, 2001, p. 41)

The preferences of patients and caregivers in different health care settings have also been explored. Consistently, the most important characteristics of CCC are described as information provision, good communication, accessibility, continuity, coordination, empowerment, and emotional support (Bechtel & Ness, 2010; Dancet et al., 2012). Several studies have also looked at the roles that clients would like to assume in the interaction with health care providers (HCPs), and interestingly the results differed across settings and countries. On one hand, people living with a chronic condition or caring for a person with a chronic condition were interested in developing partnerships with HCPs by participating in decision making about their care (Bechtel & Ness, 2010). Similarly, clients of infertility care clinics across Europe were interested in active participation (Dancet et al., 2012). On the other hand, Aro, Pietilä, and Vehviläinen-Julkunen (2012) reported that involvement in decision making and involvement of family and friends were less important for patients of Estonian intensive care units (Aro et al., 2012). Given these variations, different models of client/patient-centered care have been developed and empirically tested in different settings and disciplines (Abley, 2012; Rathert, Williams, McCaughey, & Ishqaidef, 2015).

Few studies explore actual implementation of client-centered principles and HCPs’ experiences with these approaches (Bright, Boland, Rutherford, Kayes, & McPherson, 2012; Nieuwenhuijsen, 2009). In Canada, patient care or CCC is inherent to the health care system, being one of the main foci of every hospital and health care center’s mission statement (Government of Saskatchewan, 2008; Ontario Medical Association, 2010). Because the idea has been around for some time, it is a good time to stop and reflect on what is working and what still requires further attention.

The objective of this study was to explore current models of delivery of rehabilitation services from the perspectives of both patients and families (collectively the “client”) and health care professionals (HCPs). To develop a conceptualization of client-centered principles in stroke rehabilitation, we explored the following broad questions:

- How do patients, families, and HCPs see their role in the interaction? Are clients interested in assuming an active role?
- How do clients perceive family involvement in rehabilitation?
- What are clients’ priorities for communication with HCPs, and for information?
- What are some challenges (e.g., respecting wishes, taking preferences into account) when there are more than two parties involved, and what are the ways to deal with them?
- Do HCPs feel supported and encouraged by their superiors and colleagues when practicing client/family-centered care, and in what ways (available resources, incentives, education, etc.)?
- What pros and cons do they perceive from practicing CCC?

In our work we have defined our concept as client-centered care, where client stands for the patients and family (when the latter are involved). The broad topics for the present study were guided by the client and family-centered framework developed by the *CanChild* Centre for Childhood Disability Research (2003). The core concepts of the framework are Enabling and Partnership, Providing General Information, Providing Specific Information, Coordinated and Comprehensive Care, and Respectful and Supportive Care. The applicability of the framework for adult health care has been previously supported (Bamm & Rosenbaum, 2008; Dancet et al., 2012; Schoot, Proot, ter Meulen, & de Witte, 2005). The distinctiveness of this study involved interviewing both clients (patients and families) and HCPs of the in-patient rehabilitation units in which people were recovering after a stroke. This allowed for the input of all stakeholders to be considered when developing an understanding of processes of care as experienced from all sides of the relationship.

## Method

We conducted a grounded theory study that involved semistructured interviews over a 10-month period in 2011. We invited all patients and families receiving care for at least 2 weeks in one of four rehabilitation facilities in South-Central Ontario, Canada, to participate. Participants were excluded if they did not speak English or were diagnosed with severe cognitive impairment as assessed by the Montreal Cognitive Assessment (MoCA ≥ 19) or the Mini-Mental State Examination (MMSE ≥ 21; outcome measures routinely used by stroke centers). In cases where no cognitive score was available, the decision of eligibility was left to the judgment of the clinical staff working with the patients.

Potential participants were identified and the study introduced by a health practitioner (nurse or social worker) from the patient’s direct circle of care. The participants were selected based on the previously completed survey, the Measure of Processes of Care for Adults (MPOC-A), a measure of client-centeredness of care adapted for adult health care settings (results reported elsewhere). To represent varied perspectives, interviewees were selected based on their varied perceptions of client-centeredness as assessed by MPOC-A, ensuring variation across participants (the perspectives of people who rated the services as highly client-centered might differ substantially from those whose needs are not being met by their service providers). In addition, as the interviews progressed, we looked for clients of different ages and both genders, and clinicians from different disciplines (theoretical sampling; Strauss & Corbin, 1998). A research assistant contacted consenting participants to collect demographic and contact information. The interviews took place in a venue comfortable for participants. All but one were conducted by the first author in participants’ homes (the exception was conducted at the public library) approximately 2 to 3 months after discharge. The interviews lasted 30 to 60 minutes.

All HCPs who have been practicing in one of the four rehabilitation units for at least 3 months (to ensure familiarity with the unit culture) were invited to participate. Lunch-and-learn sessions were presented at each site to inform the clinicians about the study and invite participation, following which information packages were left in the units. Interviews were conducted at the hospitals. Similar to clients, the selection of the HCPs for interviews was done based on their responses to the Measure of Processes of Care for Service Providers working with Adults (MPOC-SP(A)), a companion measure of client-centeredness of care for clinicians.

### Analysis and Rigor

To explore processes of client–health care professional interaction in the rehabilitation settings, the Grounded Theory approach developed by Strauss and Corbin (1998) was adopted. This approach was particularly suitable to answer the questions presented by this study for several reasons. First, the phenomenon under study is the process: The desired outcome of the study is developing a clear understanding of personal, organizational, and contextual factors and the interaction among them. Second, little is known about the current state of client-centeredness in rehabilitation from either the clients’ or clinicians’ perspectives. The systematic examination of the topic and creation of the model will allow better understanding of the supports and barriers to implementation of client-centered principles in adult rehabilitation (Strauss & Corbin, 1998).

To help organize and analyze the data, the qualitative data analysis computer software package NVivo, Version 9, was used (QSR International Pty Ltd., 2011). The interviews were transcribed and two researchers (E.L.B. and N.M.) carried out initial analysis independently. We started the analysis with line-by-line reading of the transcripts and breaking the data into codes (open coding). We then compared our findings. Disagreements were resolved through extensive discussions. Then the many open codes were collapsed into categories and subcategories to begin the process of refining our data. The properties and dimensions of the categories were further developed with every new interview, helping to relate the major categories to the subcategories (axial coding). Constant comparison of the properties and dimensions of the emerging categories along with multiple viewpoints presented by patients, families, and HCPs helped us to maintain objectivity during analysis. The analytic thoughts and discoveries were recorded in the theoretic memos that also helped with further development of each category. Diagrams helped to ensure clear relationships between and among the categories and identify categories that were poorly defined. When all categories were well defined and no new concepts or dimensions were emerging, no further interviews were undertaken. Finally, the central categories were integrated to create the representation of the process or the model (selective coding; Strauss & Corbin, 1998).

In addition to following grounded theory methods noted above, rigor was also ensured through a decision trail to track changes in codes and categories over the course of the project. Also ongoing discussions with the supervisory committee offered peer review opportunities. Reflexivity through journaling was used to highlight team and professional preconceptions and their impact on the process of analysis. The results of selective coding were presented to the participants and their feedback was invited.

#### Ethical considerations

The protocol of the study was approved by McMaster University’s Research Ethics Board and all participating sites’ ethics committees.

## Results

Eight patients and four family members from four rehabilitation units were interviewed. Patients ranged in age from 19 to 86, five were women, three of four family members were spouses (two women, one man), and one was the mother of the youngest participant. Fifteen HCPs from four hospitals participated in the interviews. Several disciplines were represented: Five physiotherapists, four occupational therapists, three social workers, two nurses, and one physician were interviewed.

We focused our interviews on experiences related to the intake to rehabilitation and actual rehabilitation. Five major categories were extracted and are discussed below. The categories are supported by anonymized quotes from interviews with patients (P), family members (F), and HCPs.

### Category I: Working Toward Goals Set by the Client/Being on Common Ground

This main category is in fact a combination of several threads. Participants described the importance of *working toward client set goals* (goals set jointly by the client and the therapists). We defined *compliance* as a patient’s and family’s acceptance of treatment without them being interested in voicing an opinion (i.e., passive attitude). We also were interested in exploring whether the clients felt on equal ground with the hospital staff in making decisions—*position of power*; and how they interacted with the staff during therapy or any situation when confused or angry. Did they make themselves heard—*speaking up*?

#### Working toward client set goal

Clients and clinicians having similar goals and understandings of the outcomes have been mentioned as important aspects of health care professional/client interaction at the beginning of all interviews. Building a good rapport and working together on a goal not only improves the day-to-day experiences of both clinicians and clients but also advances clients’ outcomes: “We can’t get them motivated to participate in therapy unless we are working on something that they want” (HCP).

We talked about what my goals were and we worked towards them . . . It was very much a joint—I don’t know what the point is if the other person is not going to do what you ask them to do. If you’re not going to do the exercises. They always explained why they were doing stuff, which was important for me, and for me to be successful I had to agree to what they wanted me to do and why they wanted me to do it. (P)

Clinicians would like to work in partnerships with both the patients and families where there is mutual respect, trust, honesty, and ongoing communication. Partnerships in care also resulted in better organized and efficient care, decreasing the load and the stress on the staff. Some commented on CCC being time-consuming but described it as time well spent to ensure that engaged and educated clients will be ready for discharge with less effort and stress.

#### Compliance and position of power

Although all patients and family members felt comfortable asking questions, the majority did not consider intervening in day-to-day decisions. One couple stated,

. . . if he [patient] had wanted to ask questions, I think that would have been okay. Certainly I felt that the doctor was kind of open to, you know, questions and things. You just basically were in there with a problem and you were just complying with what was being said to do and working to get out, basically. (F)

It seems that as long as the rehabilitation plan did not clash with clients’ beliefs they would not interfere, although most clients had an impression that they would be able to challenge the staff should they feel strongly about something. This idea was confirmed in several more interviews: “I just feel they [HCP] are there and they know what is, you know, best for you” (P).

#### Speaking up

Clients also have to ask questions, and question the process; this not only keeps the therapist up to date but also helps clients to direct their own care. Clients are not only the experts on their previous conditions but also on what they need to cope and return to their life. They need to understand what is going on and provide the clinicians with information about what their needs and goals are. Clinicians highlighted the importance of clients taking charge early in their recovery, feeling that this will make it easier for them to take care of themselves after they leave the hospital: “. . . we do say to them, ‘This is your therapy so without you we wouldn’t be here. You are actually the most important member of our team’” (HCP). Some clinicians suggested that poor health literacy prevents clients from active participation, and being informed will empower the client and encourage control and participation. Some clients did not raise their voice for fear of being called noncompliant or difficult. The older clients did not want to “offend” anybody by speaking their mind:

That’s the way I approach life. I don’t speak up unless it’s really important. I want people to know that when I speak up, I really mean it. It seems to me that some people are always complaining and always saying things, you know, and the other people tune out. (P)

On the contrary, one of the younger patients felt more confident about voicing his wishes and expectations; he wanted to be involved in decisions about therapies, alternative treatment providers, and overall plans of action. However, he felt that he was being perceived as “whiny” and a “complainer.”

### Category II: Support

As the patient and the family were going through very difficult times, they needed all the support that was available not only from within the family unit but also from the staff, extended family, friends, and family doctor.

#### Family

Family involvement in care and decision making improved both patients’ and families’ experiences of rehabilitation. Especially for patients whose speech was affected (aphasia) it was very important to have somebody to voice their questions and concerns: “My husband [the caregiver]. Oh yes. He was very good at asking lots and lots and lots of questions. He was asking the questions . . . he thought he knew I wanted answers to” (P).

This incredible support comes through during the interviews with two participants affected with aphasia: In each case, the family member encouraged the participant to take the time and answer the questions, at the same time trying to guess where they were going and offer some choices, but never talking for them. One of the participants commented that being in a somewhat unstable and confused state, she was happy to have her husband on her side to help her understand and follow what was going on. Family was also providing additional care and therapy: “. . . I think between my son and the therapist, that’s when we started to see the movement coming back” (F).

Family members also reflected that being present for most of the time during the rehabilitation contributed to the positive experience that they all had with the hospital stay. They also commented on always being welcome to be there, invited to the therapy sessions and social events. Being present during the day, they were able to ask all their questions and did not require any special meetings with the staff:

I found—I know some people have complained because they don’t get answers and this and that and everything else. I think that ties in with their overall involvement. How do you get answers if you are not really there, if you’re not involved, if you don’t ask the questions? (F)

The outcomes of rehabilitation often depend on family readiness (physical, mental, and emotional) to provide support: “I find the patients that do the best are the ones who have active family member involvement all the way through . . .” (HCP). It is beneficial for the family to be present for therapy sessions to see the progress and learn how to assist the patient with exercises and day-to-day activities. Family also acts as a cheerleader for a patient’s successes, and becomes an active participant of the caring team. This not only gives them confidence and decreases stress but also improves family dynamics.

#### Health care professionals

Clinicians’ attitudes also helped to shape the experience for the clients. Having a positive attitude, being caring, outgoing, enthusiastic, committed, knowledgeable, and approachable were repeatedly named as very important personal attributes of the clinicians. For the most part, the experiences were very positive, with therapists and nurses creating positive and supportive environment. However, based on her experience, one participant wanted to highlight the importance of the staff focusing on clients’ abilities versus what they cannot do. Although this was her only negative encounter with the therapists, she wanted it to be heard:

I thought that [the therapist] was detrimental to my recovery. She was so negative. My very first time I met her, she sat in that chair and I was sitting there and she spent an hour talking about all the things that I could not do. My first day home from a hospital. Couldn’t ride a bike. You can’t run. You can’t do this. You can’t do that. It was because I was feeling so good about my recovery, she just couldn’t—I don’t know. Every time she came, it was so focused on what I couldn’t do instead of what I could do. (P)

Clinicians described stroke as a life-changing experience, with clients going through the steps of grieving and acceptance, and different challenges for both the patient and the family at different stages of rehabilitation. Often, it is their first experience with a major illness that adds tremendous emotional, social, and financial burdens. These can be especially difficult for younger clients who have different family roles and are still working. The clients go through learning and adjusting processes to take control over what is going to happen next. They might present as confused, impractical, or depressed.

#### Family doctor

More than half of participants would have liked to see their family doctor as part of the hospital team. They felt that having the doctor involved would not only provide additional support and information source but also improve continuity of care after discharge. One participant felt that her doctor did not have all the information about her condition and progress, and it affected her care, especially given the complications that were mostly resolved in the hospital but still required follow-up. Other participants described often not knowing what questions to ask at the time. They believed that family doctors would have been able to explain things better and describe what was going to happen next.

#### Organizational support

The context of practice was also described as important in supporting the developing model of care. All participants described their hospitals encouraging the clients and clinicians to work together in partnership. Updating hospital mission statements, educational sessions for clinicians, posters, and clients’ education are just some of the strategies used. However, there is still a need for more practical education about actual implementation of CCC principles:

I think people get [CCC] in theory. I just don’t think we all do a very good job of implementing it—like from everyone across the board versus me in physio and her in OT and him in SLP or whatever it is. (HCP)

### Category III: Communication

All participants described the importance of good communication among all the partners: the units of the hospital, the staff members, staff and clients, and also patients and their families.

#### HCPs and clients

For the most part, there was good communication when the clients were transferred from unit to unit or among the staff of the rehabilitation ward. However, when something was not done properly or recommendations were not recorded, the clinicians turned to the patients with their questions (i.e., why is the walker the wrong height, why are you taking certain medications, why is your urine a certain color, etc.):

They would ask me questions . . . And I found that very frustrating because I couldn’t answer these questions, and then it started me worrying because if they didn’t know then you know maybe something was wrong. It was everywhere. (P)

Different personalities tend to voice their concerns differently. In one instance, the patient was walking on a broken foot because the staff was not attentive to his quiet complaints. On another occasion, the patient and her husband had repeatedly requested to be seen by a doctor to address the other chronic conditions that she was managing. Clients felt that more often the staff was focused on the immediate problem (i.e., stroke), ignoring the overall package of issues that the client brought with them.

Navigating the new system was very challenging and it was important for the clients to have a person to whom they could always turn with questions and concerns. It was not necessarily a formal caseworker, but rather any clinician who had a trusting relationship and was helpful and willing to guide the client. Many continued relying on their caseworker long after being discharged from the hospital: “. . . when there are changes when you are not well, you need somebody there to lead you through. To help you realize the different changes” (P).

Clinicians described having different strategies that help them to get everybody on the same page. In general, it is important to create a supportive environment with open and honest communication, focusing on the achievements and not on negative aspects. *Education* and *information* for both the patient and the family were mentioned by all the participants as the main strategies to help them develop a clear understanding of their condition and prognosis. Other tactics included *problem solving*—breaking the long-term goal into smaller, manageable short-term goals that still work toward the client’s ultimate goal (explaining this to the client), letting the client try the activity and discuss the results, involving the family in goal setting and discussions, and working as a team to maintain consistency:

And so it’s all a form of education ultimately but try to take the patient together with their family and me to problem solve, you know, can we attain this goal? . . . let’s get your ability to sit unsupported for a little bit before we work on standing and then walking. (HCP)

#### Among HCPs

Another frequently mentioned issue was the communication between the therapists and the nursing staff about patient progress. For example, many functional achievements could have been reinforced had the nurses supported the clients in doing things the same way as they were done during therapy sessions.

#### Patient and family

Interestingly, the communication between the patients and their families was also not always successful. Some had memory or communication problems, whereas others were just confused. The spouse of one of the participants remembers,

No I would ask him, when he would say, “Oh the doctor was in today” I would say, “Well what did they say?” and he would give me a couple of things and I would say, “Well did you ask him about this and did you” and of course he forgot quite a bit. (F)

### Category IV: Information

Not surprisingly, the issue of information was identified as one of the important attributes of good quality care. The staff (mainly nurses, therapists, and social workers) was described as being the main source of information during the hospital stay. Following discharge, support groups, friends, and family also played an important part in educating the clients about treatment options and supports available in the community. Clients also described that having written information (pamphlets, handouts, and brochures) was helpful; however, it could not replace the one-on-one information provided by the clinicians. Hence, the majority of clients who were admitted to the rehabilitation unit on Friday complained about not getting enough personal information until after the weekend (because most hospitals do not have regular therapy sessions during the weekends).

All participants agreed that when a direct question was asked, clear and detailed information was provided. However, many felt overwhelmed by their condition, and did not know what questions to ask at the moment. After the fact, they thought that clinicians, having the experience, could have foreseen what information would be useful for the clients in their particular situation: “If I asked specific questions, I was always given the answer if they could, you know? That was just fine. [But] things weren’t forthcoming, I think” (P). “. . . the information was only provided if I asked” (P).

Timeliness of the information was mentioned repeatedly by the participants. They felt that the majority of information was concentrated around transition periods (intake and discharge), and they would have liked to have the information spread out over their stay. Participants were well aware that it was impossible for the clinicians to give them specific timelines of their recovery. However, having at least tentative ranges based on previous experiences would have been greatly appreciated. It would also have made planning ahead easier: “Yeah, well I would have liked to have known more or been told more there, because really a lot of the time we didn’t know what was going to happen next” (F).

Another suggestion was to provide written materials about the rehabilitation unit prior to transfer, when the client is still on the acute ward. This would give the clients more time to learn about new rules and routines, and make the transition less stressful:

I got pamphlets, but as I said, three days after I got to rehab. It would have even been nice if they’d given it to me before when they decided I was going to go. When I was still on surgery but I was going to go down to rehab. I would have read it all and known what was going on. (P)

Clients would also have liked to know more about alternative, affordable, or private treatment options both during their stay at rehabilitation and after discharge. It was especially important for clients with a specific problem that was more pronounced than others (i.e., speech impairments, physical limitations) and who wanted to supplement the amount of treatment provided by the hospital: “. . . they didn’t really give me a choice about other options available. I’m not sure if there are other options available to me” (P).

Several successful strategies to improve CCC have been introduced in different hospitals: writing a family note (a summary that is given to the family) at the family meeting, appointing a contact person/therapy leader for each client, improving continuity and coordination of care through interdisciplinary collaborations, having the same staff working with the client, providing written materials (binder or stroke passport—a booklet including all the information pertaining to the patient’s rehabilitation: that is, goals and progress, important information regarding procedures, assistance, discharge, etc.), creating flexible environments and educational sessions for patients and families, and organizing discharge.

Overall, the majority of participants felt that they were well equipped, both physically and with information prior to discharge.

### Category V: Hospital Experiences

#### Positive features

The overall hospital experiences were also mentioned by all the participants as important attributes in shaping their satisfaction with their stay. All participants reflected on the helpfulness and kindliness of the auxiliary staff (kitchen and cleaning staff, and other services) and nurses and therapists: “Cleaning staff. They always came in with a smile on their face, so that was nice” (P).

Surprisingly, over half of the participants found meals and especially the way they were provided in a common area as one of the best experiences: “Meals honestly are one of the high points of the day [laughs]” (P). They also commented on the organization of therapy sessions and the helpfulness of having the schedule written on the notice board. This allowed the clients to plan their day and also organize visits from family and friends. Some rehabilitation units also encouraged their clients to dress in their home clothes. Many participants found it very uplifting: “I liked the fact that you got dressed every day. You didn’t feel you were sick of hospital gowns [laughs]” (P).

Weekend passes were also mentioned by several clients and HCPs as an important event. It provided an anchor for both the patient and family on the progress and special needs, and highlighted the areas that needed to be addressed before final discharge.

#### Negative features

There were several areas in which clients would like to see changes. Probably one of the most frequently mentioned was *being admitted on the weekend* (Friday). Participants felt that they were just left there to fend for themselves until the Monday when the therapy resumed. This brought up another point of *worrying and uncertainty* that many participants experienced near transition times (i.e., from unit to unit, going home) and which was increased by inadequate support when the transition happened before the weekend:

The one thing I really didn’t like was the fact I was moved to that [rehab] floor on a Friday afternoon and it was such a deadly weekend, even though I had visitors for myself but there was nothing going on the weekend. It honestly was depressing. It was depressing. The second weekend it was okay because I knew what to expect but that first weekend, it was very depressing. (P)

All HCPs described that being short staffed and having to combine several responsibilities (i.e., primary contact clinician organizing discharge) do not allow them to perform to the best of their abilities, and add stress to the staff. Many felt that there was too much demand on their time: “I feel sometimes that client-centered care needs to be supported by having the appropriate amount of staff. Having the ability for a person to be able to do the productivity within a stress-free environment” (HCP).

Understaffing also results in staff having to prioritize dealing with the problematic cases, and not providing enough support and education to the clients who are “coping well.” Not having enough time with each client was also described by all as a barrier to CCC. The clinicians also wished for more flexibility in the clients’ length of stay, and felt that not having to transfer the clients to a different rehabilitation facility would have eased the stress of the adjustment for an already vulnerable population.

#### Discharge planning

Discharge can be a challenging time for the clients, so providing education and coordinating services and supports in the community are the key for stress-free experiences. Participants commented that having a designated discharge planner improves the process of transition for the clients and decreases the load on the therapists. Having a community care representative participating in planning also improves the coordination and provides bridging to the community care.

## Discussion

**Figure 1.**
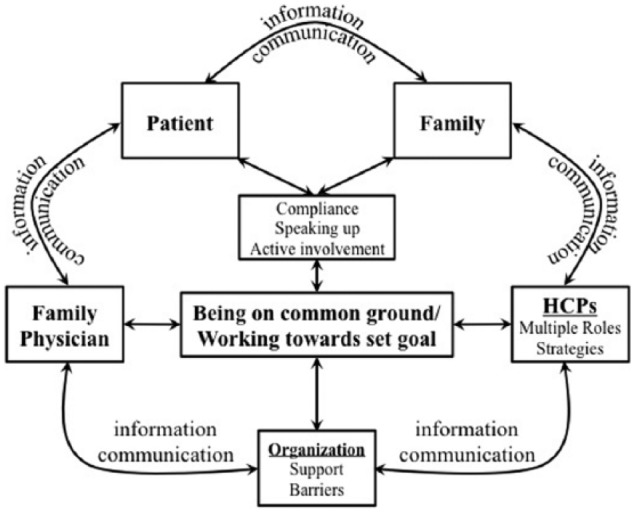
Theoretical model of processes of care. *Note.* HCPs = health care providers.

The aim of the study was to explore current client-centered processes of care from clients’ and clinicians’ perspectives using in-patient rehabilitation units as our settings. Unlike previous studies that reported some discrepancies in clients’ and clinicians’ experiences, in the current study, there were clear parallels between the identified categories that allowed creation of a uniform model (Figure 1) to describe processes of care (Berglund, Westin, Svanstrom, & Sundler, 2012; McCance, Slater, & McCormack, 2009; Rosewilliam, Roskell, & Pandyan, 2011; Tutton, Seers, & Langstaff, 2008).

The central category from both clients’ and HCPs’ perspective was the importance of the whole team having mutual goals and understanding of the outcomes: “Being on common ground” runs as the main thread throughout all the interviews. Both clients and HCPs described successful partnerships in goal setting; however, similar to other studies, the participation of the clients in other decisions, including day-to-day decisions about the amount and type of therapy, length of stay, and conditions of discharge, varied significantly based on their age and assertiveness. Older clients tended to assume a passive role, rarely voicing their opinions and preferences (Chung, Lawrence, Curlin, Arora, & Meltzer, 2012; Moreau et al., 2012), whereas younger clients faced additional challenges due to unclear diagnoses, and due to the multiple roles they were fulfilling in their prestroke lives. Although clinicians were striving to create a flexible environment, there still seemed to be set routines and procedures in rehabilitation processes. The amount of therapy could not always be adjusted to specific needs of the client. It was often limited by the understaffing of different disciplines. In addition, there were few options available to focus the treatment on specific therapy (i.e., speech language pathology or physiotherapy) for clients with severe limitations that would affect their postdischarge life. As stroke is increasingly affecting younger people (Kissela et al., 2012), the processes will have to be adjusted to better address unique needs and expectations of younger clients.

Any critical illness is a stressful, life-changing event for the entire family. It is also new ground for the clients where they might feel powerless, depressed, and unsure about any decisions. To help clients get some control over their situation, clinicians employed different strategies, including education and information provision, joint problem solving, weekend passes, and focusing on achievements. Several studies in different settings described that client education and information provision helped in setting more realistic goals for rehabilitation and also improved outcomes for both the patient and the family (Foster et al., 2012; Hunt, Moore, & Sherriff, 2012; Kergoat et al., 2012; Leach, Cornwell, Fleming, & Haines, 2010; Levack, Siegert, Dean, & McPherson, 2009). In their report on patients’ experiences on an experimental stroke unit, Lewinter and Mikkelsen (1995) described that changing the environment during weekend visits at home was having a therapeutic effect on their recovery (Lewinter & Mikkelsen, 1995).

To create productive partnerships, the importance of teamwork was highlighted by all participants. All clinicians described the patient and family as central members of the rehabilitation team. However, they would have liked the clients to be more proactive in seeking information, asking questions and participating in decision making. Most clients were happy with their role during their rehabilitation. Although they felt that they were listened to, and free to ask questions, they did not perceive themselves capable of making medical decisions. The above ideas were also described by previous studies with stroke survivors and general patients (Chung et al., 2012; Ellis-Hill et al., 2009). Similar to other studies, early family involvement was found to benefit all the team members’ experiences and outcomes (Foster et al., 2012; Levack et al., 2009; Mitchell & Chaboyer, 2010; Pellerin, Rochette, & Racine, 2011; Tutton et al., 2008). Clinicians were described as fulfilling multiple roles, advocating for clients’ best interests, educating, and providing support. Positive attitudes of HCPs were extremely important in creating pleasant experiences. Inclusion of family physicians into the rehabilitation team was seen as beneficial but was, unfortunately, not practical given the political constraints regarding hospital privileges. In the study by Wachters-Kaufmann, Schuling, The, and Meyboom-de Jong (2005), nearly half of the stroke survivors and their caregivers would prefer their general practitioners to be their main source of information, due to long-term trusting relationships and follow-up care that they provide. However, the therapists were found to provide most information (Wachters-Kaufmann et al., 2005).

Both the clients and HCPs agreed that efficient communication among all the team members and provision of timely and forthcoming information required further improvement. Importantly, these two main domains of CCC have been found deficient by previous studies (Arnold, Coran, & Hagen, 2012; Peoples, Satink, & Steultjens, 2011; Sinfield, Baker, Agarwal, & Tarrant, 2008; VisserMeily, van Heugten, Post, Schepers, & Lindeman, 2005; Wachters-Kaufmann et al., 2005). Peoples et al. (2011) conducted a systematic review of qualitative studies that explored stroke survivors’ experiences of rehabilitation. They highlighted the importance of sufficient information for improving partnerships in care, and lack of information resulting in patients’ assuming a passive role (Peoples et al., 2011).

The main barriers to implementation of CCC identified by all participants were poor health literacy, lack of time, understaffing, and organizational culture. Poor health literacy affects clients’ ability to participate actively in their care by preventing them from asking questions and making decisions. According to the Canadian Council on Learning (2007), 60% of Canadians have poor health literacy. Considering clients’ literacy and educating them accordingly is essential if active partnership in care is desired (Levasseur & Carrier, 2010).

Without exception, participants described CCC as requiring more time than medically focused care. This idea is indeed supported by the literature (Bright et al., 2012; Dilley & Geboy, 2010; Hunt et al., 2012; Leach et al., 2010; Saha & Beach, 2011). However, all agreed that it was time well spent. It allowed timely education for both the patient and family, increased clients’ participation, and consequently ownership over their condition, and resulted in improved outcomes. As stated in Bright et al. (2012), “. . . you do not have time **not** to do it” (p. 1001).

Understaffing can directly affect time available for each client, and indirectly increase stress levels and burnout of clinicians, resulting in decrease in empathy and client-centered communication (Bombeke et al., 2010; Passalacqua & Segrin, 2012). Organizational culture also has significant effects on implementation of CCC. Similar to previous studies, clinicians described the need for education, ongoing feedback, and a general organizational atmosphere that is supportive of client-centered behaviors. In turn, HCPs also reported increased job satisfaction and motivation, and better understanding of their professional identity (Dilley & Geboy, 2010; Kjörnsberg, Karlsson, Babra, & Wadensten, 2010; Perry, 2009; Rozenblum et al., 2013).

Finally, we would also like to highlight several minor, but no less important, points that were identified by the clients from different hospitals. Positive attitudes and helpfulness of the auxiliary staff (technical support, janitors, kitchen staff, etc.) made it easier to bear the long-term stay at the rehabilitation unit. Being dressed in regular clothes decreased the feelings of depression and sickness that hospital gowns often bring to people. However, being admitted on the weekend (or on Friday) left clients to adjust to the new environment without sufficient support and information from the therapists.

Some of the limitations of the current study include the relatively small number of participants. However, we felt that we gained sufficient depth and breadth in the qualitative interviews (saturation), and no new categories were emerging at the time we concluded interviewing. The study was conducted in in-patient rehabilitation units in Canada, and caution must be exercised when applying the results in different health care settings and countries. Many of the identified categories, however, were supported by international literature, and we feel that the results might be of high interest to any health care institution that has adopted, or plans to adopt, CCC as their philosophy of care.

## Conclusions and Implications

The results of the current work suggest that clinicians have a clear understanding of the principles of CCC and are working in partnerships with the clients to achieve their goals.

Regardless of age, all participants reflected on the importance of working toward goals that were meaningful and important for the client. However, some clients tend to have passive attitudes to day-to-day decisions and their preferences should be respected and supported. Clients rely on a support group that includes family, friends, staff, and family physicians. Efficient communication among all the parties is paramount. We need to encourage and educate clients to become motivated, well-informed, proactive participants in their care. Health education should begin as early as the school years to empower clients to participate in planning and decision making about their care.
